# A Case of Traumatic Tetanus

**Published:** 1881-01

**Authors:** J. W. Keene


### Clinical Reports.

### A CASE OF TRAUMATIC TETANUS.

BY J. W. KEENE, M. D.

Tuesday evening, April 6, 1880, I was summoned to attend John H. Finn, a healthy Irish lad of 16. Family history very fair, but surroundings decidedly bad. Dr. E. E. Storck was found in attendance, and at his instance I had been sent for. We continued to attend the case together until its termination. The patient had been caught by the fingers of the right hand in the heavy cog-wheel gearing in the foundry where he was employed. The fore-arm had been drawn into the machinery, and badly crushed up to the elbow. The patient thought the belt was thrown off by the accident in some way as the machinery stopped. The arm could be extricated only by removing the bolts which secured the different parts. It was said that fully fifteen minutes were thus occupied; still the prostration was slight and the shock not severe. Pulse, breathing, temperature and surface showed no marked deviation from normal type. Examination of the injured member revealed the following facts: the index finger was crushed and hanging by a portion of integument; the middle finger was stripped of soft tissues, and the bones shattered. On the wrist anteriorly was a transverse wound of the soft tissues apparently caused by one of the cogs; posteriorly a similar wound appeared a little higher up the arm, and tendons were found torn asunder. Below the elbow on the anterior aspect of the limb there was a widely gaping wound of the integument which began at the outer border of the arm and extended spirally downward and inward to the ulnar border. The integument was loosened over the anterior surface of the fore-arm, so that the examining finger passed readily under it in every direction, and emerged at the wound in the wrist before described. A compound comminuted fracture of the ulna near the middle was found; the soft parts were badly lacerated and contused; the muscles were separated from each other, and their bellies protruded from the wound in the integument. The elbow joint was intact. There were some abrasions and contusions above the elbow, but nothing of moment. The radial artery was uninjured, and the ulnar was found later pulsating normally.

With this extensive laceration and contusion of soft parts, the ulna fractured and comminuted in an open wound, the loss of the first and second fingers an assured fact, and with the likelihood of large sloughs occurring from the devitalized condition of the parts, it appeared nearly hopeless to attempt to save the limb. On the other hand, the loss of blood had been slight: circulation and innervation of the thumb and third and fourth fingers seemed perfect; the patient was young and healthy and, as usual, preferred death to amputation; the parents wished the limb preserved, if possible. It was decided in the interests of conservative surgery to attempt its preservation.

The boy and his parents were informed of the dangers of this course, that amputation might finally become unavoidable, and that at best the arm and hand would be a maimed and crippled affair.

Chloroform was administered. The metacarpal bones of the index and middle fingers were now found to be fractured near the middle of the shaft. The two fingers were accordingly removed at that point, the ends of the upper fragments squared with the bone cutters and the parts approximated by deep, firm sutures, so as to form a hand consisting of thumb, third and fourth fingers.

A splinter of bone was removed from the fractured ulna and a large fragment was found loosened, but not detached. This was, therefore, allowed to remain in hope that it might re-unite. An effort was made to approximate the ends of the fragments of the ulna with but partial success as they failed to remain in line.

The integumentary wound of the forearm was drawn together by sutures as far as possible, but a space, varying from threefourths of an inch to an inch in width, was left between the edges and through this space the muscles protruded in a hernia-like manner.

The arm was placed on a pillow and kept wet with a solution of carbolic acid and glycerine, equal parts, one dram to a pint of water. Morphiæ sulphas was ordered to relieve pain and insure sleep. The anæsthetic was well borne.

Next day, April 7, the tongue was clean, pulse slightly quickened and the temperature so little increased that observation with the thermometer was not taken. The patient slept but little, as he preferred to bear the pain which was not especially severe rather than to take the sedative prescribed. There was comparatively little swelling.

The case progressed favorably during the next few days. The patient's appetite was good, bowels regular, tongue clean. He slept fairly and made but slight complaint of pain. The fingers were drawn up by the flexors, the tendons of the extensors having been severed in part by the injury, and in the ends of these where they rested on the pillow which supported the arm he located the pain he felt.

The stitches were removed from the hand on the fifth day and the union was firm. To relieve the pain in the fingers and to facilitate dressing of the open wounds, which were now suppurating freely the arm, was suspended in a swinging apparatus, April 11, five days after the injury, and gave no further pain.

All was favorable until April 14, when he complained of a jerking in the abdomen and difficulty in moving his jaw. Temperature and pulse were normal, and the arm was doing well. April 15, marked symptoms of tetanus were present. Tonic spasms of the muscles of the abdomen, face and neck occurred with great frequency. The pulse rose to 110, the temperature became elevated but could not be taken on account of the frequency of the spasms; the tongue was heavily coated and the breath fetid; but the arm showed no change for the worse.

Chloral hydrate and bromide of potassium were prescribed in doses of gr. v of each every three hours, and in the afternoon gr. x every hour.

Dr. Briggs was called in consultation. It was decided to give strychnia a trial on the next day unless the present treatment proved successful.

April 16, 10 A. M. Much worse. Opisthotonos manifest in a moderate degree. Body can be lifted by the head and heels. Head drawn far back and immovable. Eyes fixed and staring. Spasms occur every moment. Unable to swallow. Wishes his position changed constantly. Has taken no food for twenty-four hours. Rectal feeding directed—milk and whiskey with gr. xv of chloral hydrate and an equal amount of bromide of potassium every hour. Began strychnia treatment. Gave gr. 1-32 every two hours, subcutaneously. Was seen every second hour during the day and evening by Dr. Storck or myself, or by both together. Worse at each visit.

12 M. Pulse 120; no improvement. Injected strychnia.

2 P. M. Continued treatment.

4 P. M. No improvement. Continued treatment.

6 P. M. Still worse. Gave morphiæ sulphatis gr. ½, subcutaneously.

8 P. M. Slept twenty-five minutes after injection of morphia, or at least was quiet for that time. Injection repeated. Strychnia discontinued. Left with direction to give opii tincturae ʒ ii hourly with the rectal injections.

Visited by Dr. Storck at 11 P. M. The laudanum not given as parents thought him dead at 9 o'clock. Pupils contracted to pin-head size. Low delirium. Mutters, "Boys, keep away from that machinery," etc. Sinking rapidly. Comatose when left by Dr. Storck at 12 o'clock. Can be roused by a touch of cold water, which promptly produces a convulsion.

April 17. Died at 6 A. M.

